# Dissociable Effects of Verbalization on Solving Insight and Non-Insight Problems

**DOI:** 10.3390/jintelligence13030036

**Published:** 2025-03-12

**Authors:** Laura Macchi, Francesco Poli, Laura Caravona

**Affiliations:** 1 Department of Psychology, University of Milano-Bicocca, 20126 Milan, Italy; 2 Donders Institute for Brain, Cognition and Behaviour, Radboud University, 6525 XZ Nijmegen, The Netherlands

**Keywords:** insight problems, non-insight problems, verbalization, unconscious analytic thought

## Abstract

While there is broad consensus that non-insight problems are typically solved through conscious, stepwise processes, the mechanisms underlying insight problem solving remain under debate. According to the *special process* view, insight relies on an unconscious restructuring that is susceptible to verbal overshadowing. In contrast, the *business-as-usual* approach maintains that insight and non-insight solutions both emerge via similar, conscious procedures that should be unaffected by verbalization. A third, challenging, perspective, the *unconscious analytic thought* approach, claims that the insight problem-solving process is not only unconscious but also analytic, instead of being merely associative. Actually, this process requires cognitive resources also works at an unconscious layer, suggesting that it can be disrupted by forced verbalization, which demands great cognitive effort. Therefore, according to this approach, being asked to verbalize the simultaneous processing of insight problem solving would hampers restructuring. To disentangle these positions, we compared participants’ performances on an insight problem and a non-insight problem under either concurrent verbalization or silent conditions. Our results show that verbalization significantly hampered insight problem solving, yet dramatically aided non-insight performance. Overall, our results provide evidence supporting the role of unconscious analytic processes in the resolution of insight problems, in contrast with the stepwise, conscious procedure used for the resolution of non-insight problems.

## 1. Introduction

Several lines of research have attempted to explain the process responsible for solving a well-known class of problems defined as *insight problems*. Most approaches in the literature agree that non-insight problems can be solved through a continuous step-by-step process based on trial and error; however, there is no concordance among these theories for insight problems (Gilhooly et al. 2018), in particular between the *special process* view (e.g., DeCaro and Wieth 2016; Jung-Beeman et al. 2004; Kershaw and Ohlsson 2004; Knoblich et al. 1999, 2001; Öllinger et al. 2006; Schooler et al. 1993) and the *business-as-usual* approach (e.g., Chronicle et al. 2004; Chuderski and Jastrzębski 2018; Danek et al. 2016; Fleck and Weisberg 2004, 2013; Kaplan and Simon 1990; Perkins 1981; Stuyck et al. 2022; Weisberg 2006, 2015), to such an extent that some researchers even consider the traditional classification of problems into insight and non-insight categories irrelevant (i.e., Salmon-Mordekovich and Leikin 2023).

The *special process* view argues that insight problems are solved through restructuring. This requires a period of incubation, during which a change in the representation of the problem must occur in order to find the solution to the problem. This process is considered as being mainly unconscious, which makes it unreportable and hence can only be described *a posteriori*. Conversely, the *business-as-usual* approach holds that the same incremental sequence of steps that concur in the solution of non-insight problems can be applied to insight problems. In this case, when the initial representation of the problem leads to a dead-end, and the adopted strategy does not succeed in solving the problem, a conscious analytic stepwise process reorganizes data and searches for a new strategy to be applied until the solution is reached. According to this approach, verbally reporting the procedure adopted does not affect performance on either insight or non-insight problems.

Investigating verbalization effects could help clarify the processes responsible for solving insight and non-insight problems. However, only a few authors have applied this paradigm to insight problems: Schooler et al. (1993), Ericsson and Simon (1984), Fleck and Weisberg (2004, 2013), Gilhooly et al. (2010), Macchi and Bagassi (2012), and Ball et al. (2015). We illustrate these studies and their theoretical background below, after which we introduce the theory of *unconscious analytic thought* (UAT, Macchi and Bagassi 2012, 2015) and show how it offers a parsimonious explanation for the (apparently) conflicting effects of verbalization on insight and non-insight problem solving. Finally, we provide evidence that supports our predictions.

### 1.1. Predominantly Conscious Processes

A first series of studies, using the verbalization paradigm to investigate the process underlying the resolution of insight problems, produced evidence for the *business-as-usual* approach, accounting for predominantly conscious processes. Fleck and Weisberg (2004, 2013) and Gilhooly et al. (2010) predicted a lack of verbalization effects for insight problems, showing that their resolution does not rely on processes that are hindered by verbalization, which are considered to be more similar than different to those of non-insight problems.

In particular, Fleck and Weisberg (2013) compared the performance of participants solving insight problems while thinking aloud, following the instructions of a modified version of the think-aloud procedure of (Perkins 1981, p. 33), with the performance of a non-verbalization group. They reported no differences in the solution rates between the two conditions for any of the problems and concluded that thinking aloud had no negative effects on solving insight problems. Through the analysis of verbal protocols, their findings supported the idea that various methods are adopted to solve insight problems and that there “[…] is not a sharp distinction between solving a problem through analysis versus insight” (Fleck and Weisberg 2013, p. 436).

Gilhooly et al. (2010) provided further evidence for the *business-as-usual* approach by comparing verbal versus spatial insight and non-insight problems in concurrent direct thinking aloud and silent working conditions. Their findings showed no significant difference between insight and non-insight problem solving in the verbalization condition. Moreover, verbalizing had a certain impairing effect on spatial problems (i.e., the ‘Triangle of coins’, ‘Pigs in a pen’, ‘Necklace’ problems, etc.), while verbal problems (i.e., the ‘Lilies’, ‘Horse trading’, ‘Reading’ problems, etc.) were helped somewhat by verbalization. However, the authors admit that the process involved in restructuring under the *business-as-usual* view needs further clarification.

These results, which replicate a lack of verbalization effects in insight problem solving, go in the same direction as those reported by Fleck and Weisberg and highlight the idea that insight problem solution processes are not disrupted by verbalization, supporting the *business-as-usual* explanation. However, the effects of verbalization in insight studies reported in the literature are far from consistent.

### 1.2. Predominantly Unconscious Processes

Schooler et al. (1993), by adopting a modified retrospective verbalization procedure, collected evidence showing the interference of verbalization with the solution of insight problems. When participants were asked to write down their thoughts while solving the problem, they were less successful at solving the insight problems. In an additional experiment, the authors compared insight problems with non-insight problems, showing that while concurrent verbalization had no effect on the latter, it impaired performance in the former. These results support the authors’ hypothesis that verbalization disrupts the critical non-reportable processes that characterize insight problem solving, interfering with the successful solution of the problem. The authors stated that “with insight problems, a request for verbalization may cause the verbalizable task components to overshadow those that are less readily verbalized” (Schooler et al. 1993, p. 178). The solution to insight problems occurs suddenly (Metcalfe and Wiebe 1987), thus suggesting that the critical steps leading to the solution are unavailable for conscious inspection. Moreover, although it is quite possible that participants think aloud when solving insight problems, it seems that it is not the reportable process that solves the problem (Schooler et al. 1993).

Conversely, online verbalization is typically benign in reaching the solution of non-insight problems (Ericsson and Simon 1984) because this type of problem requires a completely conscious, and hence reportable, procedure. Overall, the absence of a verbalization effect on non-insight problems and its effect on insight problems seems to point to the existence of a different process for the two categories of problems. However, the nature of these processes still needs to be specified (Schooler et al. 1993).

Ball et al. (2015) also investigated the presence of verbal overshadowing in insight problem solving, with the specific aim of verifying whether reducing opportunities for speech-based processing could facilitate reaching the solution. The authors support the idea that the type of *special process* that leads to overcoming the impasse occurs at an unconscious (and therefore non-reportable) level through problem restructuring (e.g., Bowden et al. 2005; Gilhooly et al. 2018; Ohlsson 1992, 2011), resulting from the facilitative effect of distracting internalized discourse-based thought processes. More effective unconscious, unreported thought processes would emerge, allowing weakly activated solution concepts to strengthen and spill over into conscious awareness.

To investigate this aspect of verbalization, the authors introduced a condition in which their participants were asked to think aloud while working on visuo-spatial insight problems. As predicted, their performance suffered, with results comparable to a silent working (control) condition. Conversely, the articulatory suppression condition (in which one speaks aloud a stereotypic number sequence repeatedly) and the irrelevant speech condition (in which one listens to a looped sequence of digits spoken in a monotone voice) produced a greater insight problem-solving effect. According to the authors, reducing speech-based processing allows more relevant processing to take place at an unconscious level. In particular, distraction resulting from articulatory suppression and irrelevant speech would have a favorable effect on insight problem solving due to its disruptive effect on internal discourse in terms of reducing opportunities for speech-based problem processing.

On the other hand, our view is based on the hypothesis of *unconscious analytic thought*, formulated by (Macchi and Bagassi 2012; Bagassi and Macchi 2016). The main idea of the authors is that the reinterpretation of the critical point in insight problems is guided by a goal-oriented, unconscious, and analytic process. According to this approach, “the way of thinking involved in insight problem solving is very close to the process involved in the understanding of an utterance, when a misunderstanding occurs” (Macchi and Bagassi 2015, p. 147). To resolve the misunderstanding, a more appropriate meaning must be selected and the default interpretation—which led to a “fixation”—has to be discarded in order to “restructure” the problem. The aim of this process is to grasp the fundamental characteristics of the problem structure (i.e., the ones that are more relevant to its aim and to the context), leading to a parallel search informed by relevance at the unconscious level. Verbalizing while attempting to solve the problem encourages the use of conscious processes and hence tends to fixate the problem solver and hinder the restructuring process (Macchi and Bagassi 2012).

On the other hand, our view is based on the hypothesis of *unconscious analytic thought*, formulated by (Macchi and Bagassi 2012; Bagassi and Macchi 2016). The main idea of the authors is that the reinterpretation of the critical point in insight problems is guided by a goal-oriented, unconscious, and analytic process. According to this approach, “the way of thinking involved in insight problem solving is very close to the process involved in the understanding of an utterance, when a misunderstanding occurs” (Macchi and Bagassi 2015, p. 147). To resolve the misunderstanding, a more appropriate meaning must be selected and the default interpretation—which led to a “fixation”—has to be discarded in order to “restructure” the problem. The aim of this process is to grasp the fundamental characteristics of the problem structure (i.e., the ones that are more relevant to its aim and to the context), leading to a parallel search informed by relevance at the unconscious level. Verbalizing while attempting to solve the problem encourages the use of conscious processes and hence tends to fixate the problem solver and hinder the restructuring process (Macchi and Bagassi 2012).

Reflective and conscious processes cannot guarantee the solution to insight problems (differently from the *business-as-usual* approach), since the solution can be reached only when the impasse is overcome by restructuring, and an “insight” emerges. The insight problem solution is achieved by productive and creative thinking, which is derived from covert and unconscious thinking processes, including relevant, analytic, and goal-oriented activity that goes beyond random associative, as claimed by the *special process* view. During this wide-ranging search, verbalization would act as a constraint and a hindrance to finding the still-elusive solution, which is partly unconscious and non-reportable. Thinking works with continuous, implicit, unconscious processes, which cannot be reported while verbalizing.

This hypothesis was confirmed by the study carried out by Macchi and Bagassi (2012), in which they investigated whether serial online verbalization disrupts the solution process for insight problems. The authors showed that, with both the insight problems that were employed (i.e., the ‘Pigs in a pen’ and the ‘Square and parallelogram’ problems), a significantly smaller percentage of participants solved the insight problem in the verbalization condition in comparison to the non-verbalization condition. These results highlight that concurrent verbalization hinders insight problem-solving processes, supporting the idea that this type of problem cannot be solved through step-by-step incremental processes, but rather by unconscious thought processes.

It is important to keep in mind that these observations only apply to concurrent online verbalization and not to all language-related tasks that may be carried out during the processing of a problem (i.e., spontaneous verbalization did not impair the insight problem solving, see Macchi and Bagassi 2012). If we consider the task that Ball et al. (2015) used, i.e., the concurrent articulation of a stereotypic number sequence whilst tackling the insight problem, it is easy to see that even if verbalization is involved, it is not effortfully demanding. In fact, this latter activity was seen to facilitate insight problem solving.

Conversely, the type of online verbalization we used in our study can be considered highly demanding as it not only requires constant attentional focus on the conscious layer, but also greater cognitive effort in comparison to the concurrent articulation of a stereotypic number sequence and spontaneous thinking aloud. Moreover, fatigue is increased by the fact that this externalization of thought is not spontaneous but is explicitly required and continuously solicited, which contrasts with and hinders the unconscious processing necessary for restructuring. In this sense, forced simultaneous verbalization, according to our hypothesis, prevents thought processes that are not only unconscious but also high-quality (UAT).

In a previous study on incubation (Caravona and Macchi 2022), participants carrying out a *high-demanding incubation task* (e.g., a crossword puzzle or Sudoku) while working on an insight problem performed worse than those doing a *low-demanding incubation task* (e.g., a routine one/three-digit arithmetic task). These results highlight that insight problems are solved at an unconscious level, which—if success is to be attained—must not be overloaded with cognitive requests during the incubation phase (induced or spontaneous). This is to be attributed to the fact that this high-demanding task requires attentional focus at a conscious layer and cognitive resources at both the conscious and unconscious ones, leaving no resources free to process, unconsciously, the insight problem in the given time^1^.

Similarly, being asked to verbalize the simultaneous processing of an insight problem solution not only requires attentional focus but also fully engages cognitive resources; hence, concurrent verbalization appears to have similar characteristics to the *high-demanding task* described above, being fully conscious and demanding great effort, thus impeding the exploration of different ways to process the task and the request.

This hypothesis attempts to answer the issue about the relation between verbalization and insight processes: “although […] verbalization overshadows critical difficult-to-report insight processes, the nature of these processes remains to be specified” (Schooler et al. 1993, p. 178).

### 1.3. Controversial Effects of Verbalization

Before introducing the study we are presenting in this paper, it is worth commenting on the different effects of verbalization found in the studies cited above, as a number of them reported that it had a disruptive effect on insight problems (Ball et al. 2015; Macchi and Bagassi 2012; Schooler et al. 1993), while others did not (Fleck and Weisberg 2004, 2013; Gilhooly et al. 2010).

It is difficult to identify the reason for the conflicting verbalization effects since there are no specific differences in the experimental design adopted by the studies supporting the different approaches (*business-as-usual*, *special process* and UAT). However, there are differences between the specific studies, mainly in the number of problems used and the time provided to solve them: Gilhooly et al. (2010) submitted 32 problems, including insight and non-insight problems, while Macchi and Bagassi (2012) and Ball et al. (2015) used 2 and 3 insight problems, respectively. The time available to solve each problem also varied greatly between experiments, ranging from four minutes (Gilhooly et al. 2010) to ten minutes (Fleck and Weisberg 2004, 2013) per problem.

There were also differences in how the results were analyzed: Gilhooly et al. (2010), Fleck and Weisberg (2004, 2013), and Macchi and Bagassi (2012) reported the effects of verbalization for every single problem, while the other studies only provided the combined result of all the insight problems considered. Additionally, some of the studies only used insight problems (Ball et al. 2015; Fleck and Weisberg 2004, 2013; Macchi and Bagassi 2012), while others used both insight and non-insight problems.

**Figure 1:**
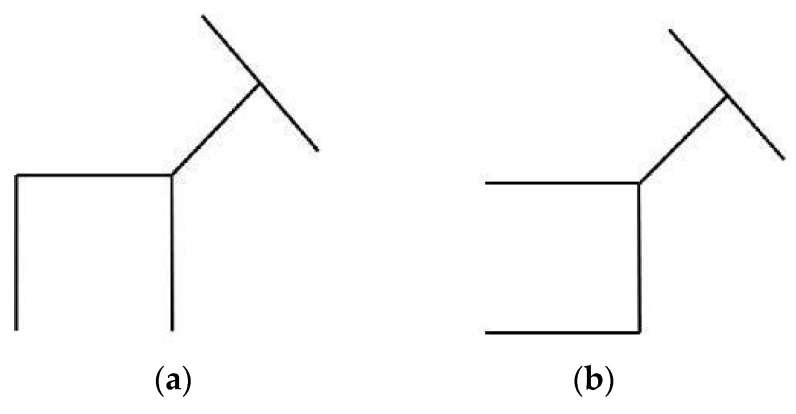
(**a**) The Deer problem; (**b**) The Deer problem solution.

**Figure 2:**
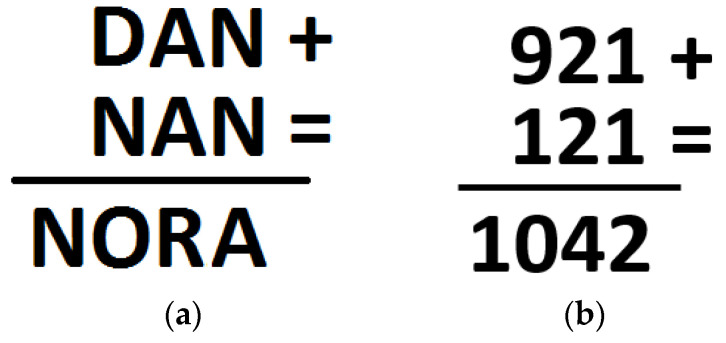
(**a**) The Revised cryptarithmetic problem; (**b**) The Revised cryptarithmetic problem’s solution.

We avoided confusion between all the variables that could account for the presence or absence of the verbalization overshadowing effect by only including one insight problem and one non-insight problem in our study, presenting them to two different, but equivalent, groups of participants. This also allowed us to make a direct comparison between problems without including potentially influencing variables (e.g., within-participant effects due to working on both insight and non-insight problems, the order in which the problems were presented, and the number of problems submitted). Moreover, in our view, the time needed to work on an insight problem depends on its level of difficulty and varies from one problem to another. We used a pilot study to estimate the time needed for the problems adopted in this study (the ‘Deer problem’ and the ‘Revised cryptarithmetic problem’, reported in Figure 1a,b and Figure 2a,b), calculating ten minutes as being adequate.

## 2. Experiment

The present study, which follows the UAT line of research, aims to replicate the results obtained by Macchi and Bagassi (2012) with a different insight problem, but it is also designed to examine the effect of verbalization on non-insight problems. According to our hypothesis, as the solution process in insight problems is mainly unconscious, concurrent online verbalization would have the effect of preventing it from developing, thus compromising the possibility of reaching the correct solution. Moreover, we claim that this type of verbalization, due to its characteristics of great cognitive effort and attentional focus requested would act similarly to a high demanding incubation task impeding the analytical process necessary in the resolution of insight problems to develop. However, in the case of non-insight problems, the solution process would be conscious, serial, and stepwise; hence, as already demonstrated by Ericsson and Simon (1984), concurrent verbalization would be beneficial in elaborating and organizing it. However, the particularity of the present experiment is that it allows for a comparison of the effects of verbalization on the two different types of problems, with the same controlled methodology.

## 3. Methods

### 3.1. Participants and Procedure

Our study involved 120 undergraduate students of the University of Milano-Bicocca. Participants were aged between 19 and 25 years. Each participant was tested individually.

Half of the participants were given an insight problem (the ‘Deer problem’) and the other half a non-insight problem (the ‘Revised cryptarithmetic problem’). Two conditions were created for each problem, a *verbalization* condition and a *non-verbalization* condition, with each participant being randomly assigned to one of them (N = 30 for each condition).

In the verbalization condition, participants were instructed to verbalize while they were carrying out the problem, as required by the standard think-aloud procedure (Ericsson and Simon 1984), and hence to explain the procedure they were adopting. If they were silent for longer than 15 s, they were reminded to think out loud. They worked on the problem until they reached the solution or gave up. Conversely, in the non-verbalization condition, participants were asked to remain silent while solving the problem. The time taken to solve the problem or quit was recorded for each participant. The time limit to consider correct responses as valid was set at 10 min.

### 3.2. Materials

The insight problem adopted in our experiment was the ‘Deer problem’ (see Figure 1a,b).

Imagine that this is a deer. It is looking to the right. Your task is to change the position of one of these 5 lines so that the deer is looking in another direction.

The non-insight problem used was a simplified version of the classical cryptarithmetic problem, shown in Figure 2a,b.

It is necessary to replace each letter with a number in order to obtain a correct arithmetic result, knowing that a number from 1 to 10 corresponds to a letter, and each letter must be replaced by a different number from the one used for any other letter.

### 3.3. Analysis

We adopted a Cox proportional hazards regression model (Cox and Oakes 1984) to examine how verbalization (present or absent), problem type (insight or non-insight), and their interaction influenced the likelihood of reaching a solution over time. The Cox model is well suited to time-to-event data, where the “event” is problem solution. In our study, the time variable was the number of seconds elapsed from the start of the problem until the participant solved the problem (i.e., the event) or until the 10 min deadline, whichever occurred first. This approach allowed us to account for the fact that not all participants solved the problem and that the time to solution varied from one individual to another.

A key advantage of the Cox model is that it does not assume a particular underlying distribution of survival times and instead uses a semi-parametric approach (Wong et al. 2017). Additionally, the Cox model handles right-censored data—in our case, participants who reached the time limit without solving the problem. This feature is especially important given that an imposed time limit can distort the distribution of times to solution. The Cox model’s partial-likelihood approach adjusts for this bias, providing robust estimates of the effects of our independent variables on the success rate. We implemented the model using the coxph function from R’s survival package (Therneau 2024).

## 4. Results

The proportions of correct solutions for the insight and non-insight problems across verbalization and non-verbalization conditions are reported in Table 1.

**Figure 3:**
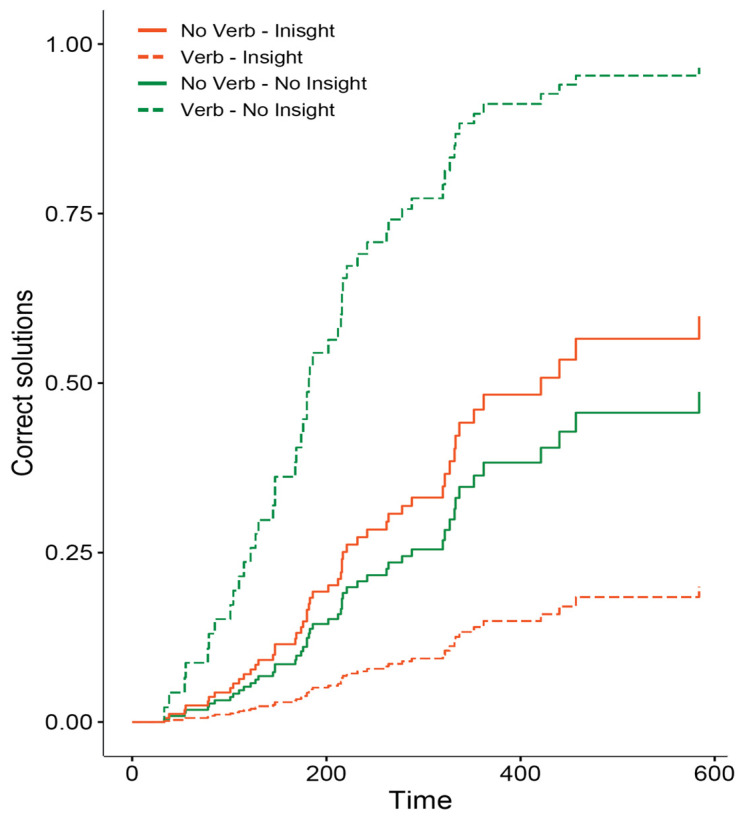
Survival curves calculated based on Cox regression model separately for each condition.

Next, we report the results of the Cox regression model, which tested differences in the time taken to achieve the correct solution across problem type (insight and non-insight) and verbalization (present and absent). We found a significant main effect of verbalization on success rate (z = 4.4, β = 1.76, SE = 0.4, *p* < 0.001, e^β = 5.79, 95% CI = [2.65, 12.67]), no main effect of problem type (z = 0.97, β = 0.41, SE = 0.4, *p* = 0.33, e^β = 1.51, 95% CI = [0.65, 3.49]), and a significant interaction effect (z = −4.6, β = −3.17, SE = 0.70, *p* < 0.001, e^β = 0.04, 95% CI = [0.01, 0.16]). The Cox model results are reported in Figure 3.

## 5. Discussion

Our results showed that participants in the verbalization condition were significantly less successful in solving the insight problem than those in the non-verbalization condition. Indeed, only 13% of the participants solved the problem while verbalizing, compared to 57% in the silent condition. Conversely, in the case of the non-insight problem, 80% of the participants were successful in solving the problem in the verbalization condition, but only 30% in the non-verbalization condition.

These results indicate that the process responsible for solving insight problems is different from that of solving non-insight problems. Concurrent online verbalization compromised the achievement of the solution of the insight problems, while it helped the process in the non-insight problems. This kind of verbalization actually seems to be beneficial in processing and organizing the solutions of non-insight problems, which rely on conscious stepwise processes. On the other hand, it seems to disrupt the solution of insight problems as they largely rely on unconscious processes. While working on the solution of an insight problem, an increasingly relevant analysis of the characteristics of the elements involved is taking place in order to find a new interrelation between them. In this stage, a new representation of the problem still has to be discovered so the problem solver is unable to verbally express and describe the procedure. Indeed, verbalization acts as a constraint in this phase of a wide-ranging search in which the solution has yet to be found and so impairs the process. Since the concurrent verbalization procedure encourages conscious processing of the problem, it prevents the search process, which is at least partly unconscious and therefore cannot be described while it is in progress.

With respect to the diatribe between predominantly conscious vs. unconscious processes, our results would seem to support the latter, as we found different effects of verbalization with insight and non-insight problems, not supporting the *business-as-usual* approach. Furthermore, our findings suggest that the insight problem-solving process should not be considered as merely associative unconscious (*special process* views) but as an *unconscious analytic thought*. As previously mentioned, online verbalization is conscious and effortful, impeding the exploration of different ways to understand the insight problem (in accordance with Macchi and Bagassi 2015) and thus playing a similar role to the *high-demanding tasks* in the incubation manipulation carried out by Caravona and Macchi (2022). Being asked to articulate a thought related to the simultaneous processing of a problem not only requires attentional focus but also fully engages cognitive resources. By verbalizing the resolution process of the insight problems, participants were stuck and unable to apply new representations of the problem.

The overall results that emerged from our study could certainly be explored further by future studies with more direct measures, such as behavioral measures. Several studies have for instance investigated the solution behaviors activated by participants during the solution process to examine solution strategies or analyze solution behaviors in problem solving (Bianchi et al. 2020; Fedor et al. 2015; Danek et al. 2016).

There may also be several practical implications of our results that warrant further investigation, one of which is certainly their relevance to the field of education. In this context, reflective, conscious thinking—undoubtedly crucial for learning—is often overemphasized and regarded as the only valid way to process data and information. Meanwhile, for creative thinking, forms of implicit, unconscious thinking could be highly beneficial.

## Figures and Tables

**Table 1:** Proportion of problem solution across conditions.

	No Verbalization	Verbalization
Insight problem	57%	13%
Non-insight problem	30%	80%

## Data Availability

The data and code are fully available on the accessible repository OSF at the following link: https://osf.io/zar73/?view_only=1a968bd6aba446089640e061b69b6688 (accessed on 27 January 2025).

## Note

1 On the contrary, the *low-demanding task* required only a routinary mechanical procedure, leaving cognitive resources available at the unconscious level to solve the insight problem. This task resulted in the best performance (response rate and resolution time), thus confirming that insight problem resolution depended on an unconscious and analytical process.
